# Supplementary material for: Imaging gigahertz zero-group-velocity Lamb waves
Source: Nat Commun. 2019 May 20;10:2228. doi: 10.1038/s41467-019-10085-4 (PMC6527571; doi:10.1038/s41467-019-10085-4)
Supplement: Supplementary file 1 — Supplementary Information [file 41467_2019_10085_MOESM1_ESM.pdf]

Supplementary Information
Imaging Gigahertz Zero-Group-Velocity Lamb Waves

Mezil et al.

SUPPLEMENTARY NOTE 1: THEORETICAL MODEL

The geometry is shown in Supplementary Fig. 1. We assume that the two layers are isotropic, homogeneous and infinite, with mass density ρ_i, longitudinal and transverse velocities v_{Li} and v_{Ti}, and thicknesses h_i, where $i = 1, 2$ indicates the layer number. The coupling between the layers is taken to be perfect, i.e., by assuming continuity of the displacement and stress components at the interface ($z = 0$). The ω (angular frequency) – k (wavenumber) relation is solved using the scalar potential ϕ and the vector potential ψ, where the latter is reduced to a scalar as the problem is two-dimensional. The tangential and normal displacements are derived from these potentials as follows:

$$u_x = \frac{\partial \phi}{\partial x} - \frac{\partial \psi}{\partial z}, \qquad u_z = \frac{\partial \phi}{\partial z} + \frac{\partial \psi}{\partial x}, \tag{S1}$$

and the stresses are given by

$$\sigma_{xz} = \mu \left(\frac{2\partial^2 \phi}{\partial x \partial z} + \frac{\partial^2 \psi}{\partial x^2} - \frac{\partial^2 \psi}{\partial z^2} \right), \tag{S2}$$

$$\sigma_{zz} = \lambda \left(\frac{\partial^2 \phi}{\partial x^2} + \frac{\partial^2 \phi}{\partial z^2} \right) + 2\mu \left(\frac{\partial^2 \phi}{\partial z^2} + \frac{\partial^2 \psi}{\partial x \partial z} \right), \tag{S3}$$

where λ, μ are the Lamé coefficients[1].

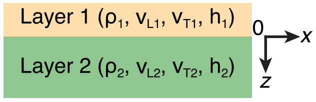

Supplementary Fig. 1. Geometry of the bilayer model.

The potentials in the layers can be expressed as

$$\begin{cases} \phi_1 = [A_{1L} \cos(p_1 z) + B_{1L} \sin(p_1 z)]\, e^{i(kx-\omega t)} \\ \psi_1 = [A_{1T} \cos(q_1 z) + B_{1T} \sin(q_1 z)]\, e^{i(kx-\omega t)} \\ \phi_2 = [A_{2L} \cos(p_2 z) + B_{2L} \sin(p_2 z)]\, e^{i(kx-\omega t)} \\ \psi_2 = [A_{2T} \cos(q_2 z) + B_{2T} \sin(q_2 z)]\, e^{i(kx-\omega t)}, \end{cases} \tag{S4}$$

where p and q are the z-components of the longitudinal and transverse wave vectors, respectively. The wavenumbers $k_{Li} = \omega / v_{Li}$ and $k_{Ti} = \omega / v_{Ti}$ satisfy dispersion relations for bulk waves ${k_{Li}}^2 = k^2 + {p_i}^2$ and ${k_{Ti}}^2 = k^2 + {q_i}^2$. A_{iL}, B_{iL} are the amplitudes of longitudinal components and A_{iT}, B_{iT} are the amplitudes of shear components.

At the free boundaries ($z = -h_1$ and h_2), the stresses normal to the surface (σ_{xz} and σ_{zz}) vanish, whereas at the interface ($z = 0$), the continuity of displacement and stresses is applied. It follows that

$$\begin{cases} \sigma_{zz1} = \sigma_{xz1} = 0 & \text{for } z = -h_1, \\ \sigma_{zz1} = \sigma_{zz2} & \text{for } z = 0, \\ \sigma_{xz1} = \sigma_{xz2} & \text{for } z = 0, \\ u_{x1} = u_{x2} & \text{for } z = 0, \\ u_{z1} = u_{z2} & \text{for } z = 0, \\ \sigma_{zz2} = \sigma_{xz2} = 0 & \text{for } z = h_2. \end{cases} \tag{S5}$$

From Supplementary Eqs. (S1–S5), the problem can be rewritten in matrix form, $\mathbf{M} \cdot \mathbf{U} = [0]$:

$$\begin{bmatrix} 2ikp_1 \sin[p_1h_1] & 2ikp_1 \cos[p_1h_1] & ({k_{t1}}^2 - 2k^2)\cos[q_1h_1] & -({k_{t1}}^2 - 2k^2)\sin[q_1h_1] & 0 & 0 & 0 & 0 \\ -({k_{t1}}^2 - 2k^2)\cos[p_1h_1] & ({k_{t1}}^2 - 2k^2)\sin[p_1h_1] & 2ikq_1 \sin[q_1h_1] & 2ikq_1 \cos[q_1h_1] & 0 & 0 & 0 & 0 \\ 0 & 2ik\mu_1p_1 & ({k_{t1}}^2 - 2k^2)\mu_1 & 0 & 0 & -2ik\mu_2p_2 & -({k_{t2}}^2 - 2k^2)\mu_2 & 0 \\ -({k_{t1}}^2 - 2k^2)\mu_1 & 0 & 0 & 2ik\mu_1q_1 & ({k_{t2}}^2 - 2k^2)\mu_2 & 0 & 0 & -2ik\mu_2q_2 \\ ik & 0 & 0 & -q_1 & -ik & 0 & 0 & q_2 \\ 0 & p_1 & ik & 0 & 0 & -p_2 & -ik & 0 \\ 0 & 0 & 0 & 0 & -2ikp_2 \sin[p_2h_2] & 2ikp_2 \cos[p_2h_2] & ({k_{t2}}^2 - 2k^2)\cos[q_2h_2] & ({k_{t2}}^2 - 2k^2)\sin[q_2h_2] \\ 0 & 0 & 0 & 0 & -({k_{t2}}^2 - 2k^2)\cos[p_2h_2] & -({k_{t2}}^2 - 2k^2)\sin[p_2h_2] & -2ikq_2 \sin[q_2h_2] & 2ikq_2 \cos[q_2h_2] \end{bmatrix} \cdot \begin{bmatrix} A_{1L} \\ B_{1L} \\ A_{1T} \\ B_{1T} \\ A_{2L} \\ B_{2L} \\ A_{2T} \\ B_{2T} \end{bmatrix} = \begin{pmatrix} 0 \\ 0 \\ 0 \\ 0 \\ 0 \\ 0 \\ 0 \\ 0 \end{pmatrix}. \tag{S6}$$

Non-trivial solutions are found when the determinant of the 8×8 matrix $\mathbf{M}$ vanishes, i.e., $\det(\mathbf{M}) = 0$. In order to avoid (unwanted) bulk waves propagating at velocities v_{Li} ($p_i = 0$) and v_{Ti} ($q_i = 0$), the terms p_1, q_1, p_2 and q_2 can be factorized in the 2nd, 4th 6th and 8th rows, respectively. The dispersion curves of the bilayer structure is then estimated by determining the zeros of the secular equation. As the structure is spatially asym-

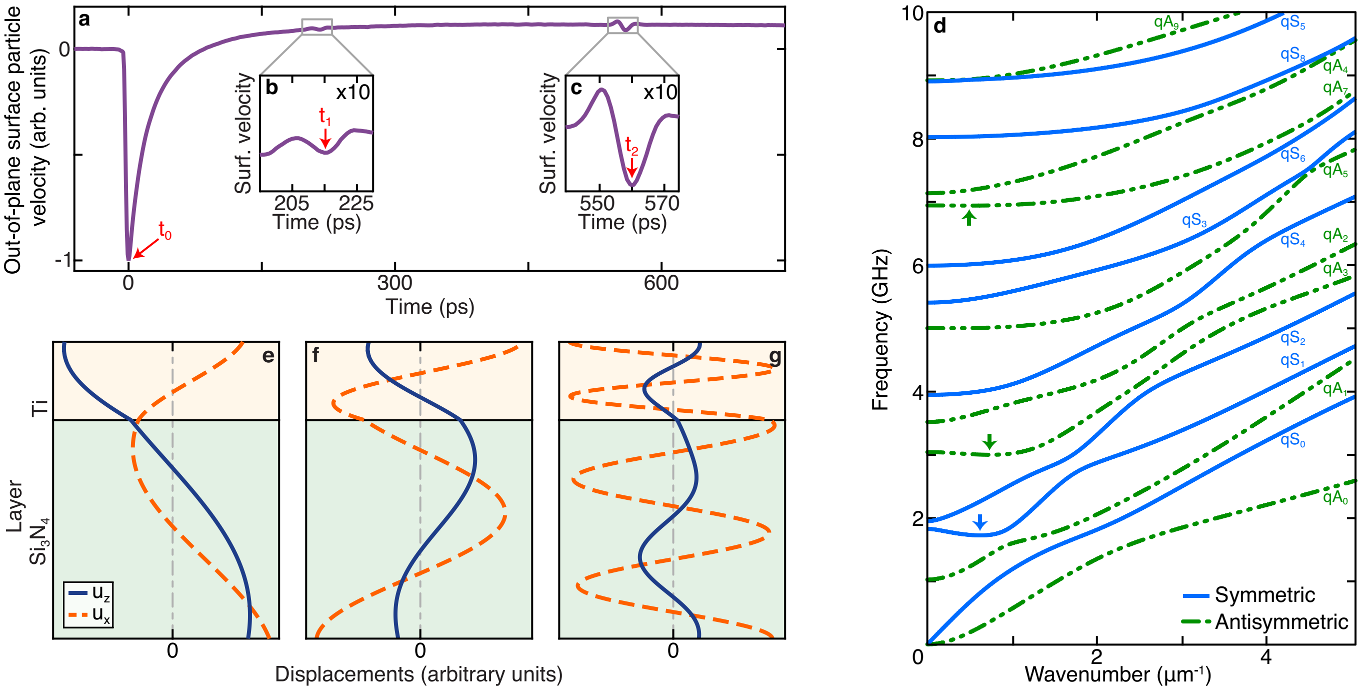

Supplementary Fig. 2. Pulse-echo measurements and ZGV mode displacements. **a** Surface particle velocity temporal variation, showing acoustic echoes. Zoom-in on the echoes from **b** the interface and **c** the rear surface of the sample. **d** Calculated dispersion curves of the bilayer system. **e–g** Calculated normal (solid line) and tangential (dashed line) displacements in the Ti/Si_3N_4 bilayer for the first three ZGV Lamb modes **e** at $f_1^{th} = 1.7248$ GHz and $k_1^{th} = 0.620$ μm^{-1} **f** at $f_2^{th} = 3.0014$ GHz and $k_2^{th} = 0.732$ μm^{-1} and **g** at $f_3^{th} = 6.9476$ GHz and $k_3^{th} = 0.492$ μm^{-1}.

metric, modes cannot be classified exactly as symmetric and antisymmetric. For a given mode, the group velocity is extracted using $v_g = \partial\omega/\partial k$. A solution (ω, k) is identified as a ZGV mode if $v_g = 0$ with $k \neq 0$. Furthermore, normal and tangential displacements—u_z and u_x, respectively—can be estimated from the dispersion curves. For a solution (ω, k), the equations representing the boundary conditions can be be solved once a component common to $\mathbf{U}$ is fixed (e.g., $A_{1L} = 1$). This gives access to the relative displacements $u_{x,z}$.

SUPPLEMENTARY NOTE 2: SAMPLE AND EXPERIMENTAL PARAMETERS

The sample consists of a silicon-nitride membrane provided by NTT Advanced Technology Corporation (MEM-N0302) with a nominal thickness of 2.0 ± 0.2 μm. It is mostly composed of Si_3N_4, but is not a pure crystal (the composition ratio Si:N is between 3:4 and 1:1). Nevertheless, it is hereafter denoted as Si_3N_4. The membrane is supported on its edges by a Si frame, providing a 3×3 mm^2 area with free surfaces, necessary to generate ZGV Lamb modes. The membrane is coated with a ~650 nm sputtered polycrystalline titanium film. To calculate the dispersion curves, the elastic constants and density are taken from Ref. [2]: $v_{L_{Ti}} = 6130$ m.s^{-1}, $v_{T_{Ti}} = 3182$ m.s^{-1}, $\rho_{Ti} = 4508$ kg.m^{-3} for titanium and $v_{L_{Si_3N_4}} = 10607$ m.s^{-1}, $v_{T_{Si_3N_4}} = 6204$ m.s^{-1}, $\rho_{Si_3N_4} = 3185$ kg.m^{-3} for silicon nitride. In order to accurately determine the thicknesses, an experiment measuring the surface particle velocity in the time domain is carried out using an interferometric pulse-echo method with focused pulsed-laser beams (~1.5 μm $1/e^2$ diameter) incident from the top side of the sample and with picosecond time resolution. The pump beam is modulated at $f_p = 1$ MHz, and we monitor the in-phase output of the lock-in amplifier. The result is shown in Supplementary Fig. 2a. The first minimum in the variation at $t_0 = 0$ is related to the temperature rise and deformation caused by the laser pulse. The echo at t_1 corresponds to the acoustic pulse reflected from the Si_3N_4/Ti interface, whereas the second echo at t_2 corresponds to the acoustic pulse reflected from the rear surface of the membrane. The weak reflection from the interface (at t_1) indicates good adhesion (as our model assumes). The corresponding time intervals are $\Delta t_1 = 215 \pm 1$ ps and $\Delta t_2 = 560 \pm 1$ ps, allowing us to evaluate the thicknesses of 659 ± 3 and 1830 ± 10 nm for the Ti and the Si_3N_4 layers, respectively, from the known v_L values. (Errors correspond to those arising from the time resolution of the apparatus.). For Si_3N_4 the thickness agrees within the 10% uncertainty given by the supplier.

The corresponding predicted dispersion curves are shown in Supplementary Fig. 2d. The mode classification follows the one suggested by Mindlin[3], where the

Supplementary Table I. First three ZGV Lamb mode frequencies f and wavenumbers k for the Ti/Si_3N_4 bilayer.

	Mode	f (GHz)	k (µm^{-1})
1	qS_1	1.7248	0.620
2	qA_3	3.0014	0.732
3	qA_7	6.9476	0.492

integers correspond to the number of antinodes of the mechanical displacement. This integer can be negative in case of negative group velocity. The 'q' denomination relates to the term quasi- in the appellations quasi-symmetric and quasi-antisymmetric, related to the sample spatial asymmetry. For the zero wave-vector modes (i.e., for $k = 0$ µm^{-1}), qA_{2n}, qS_{2n+1} have an out-of-plane displacement whereas qA_{2n+1}, qS_{2n} have an in-plane displacement. Therefore, the former are more likely to be observed in our experiments. Three ZGV Lamb modes are predicted below 10 GHz. They are then referred as qS_1, qA_3 and qA_7, and are labelled 1, 2, 3, respectively, for simplicity (see Supplementary Table I). Their frequencies and associated wavenumbers are displayed in Table I. With the arbitrary-frequency method (see Methods in the Main text), these frequencies are accessible by modulating the pump beam at the frequency $f_p = 36.8$, $f_p = 27.3$, and $f_p = 34.9$ MHz, for the first, second and third ZGV Lamb modes, respectively.

We also present the normal and tangential displacements of these three ZGV modes in Supplementary Figs 2e-g. At the top free surface, i.e., where the excitation and detection occur, the tangential displacement is significant for the three modes. Conversely, the normal displacement is different for these modes: it is predominant for the lowest ZGV mode at $f_1^{\mathrm{th}} = 1.7248$ GHz (Supplementary Fig. 2e), still significant for the second one at $f_2^{\mathrm{th}} = 3.0014$ GHz (Supplementary Fig. 2f) and relatively weak for the third one at $f_3^{\mathrm{th}} = 6.9476$ GHz (Supplementary Fig. 2g).

Finally, the pump beam radius should be carefully chosen to enhance ZGV Lamb mode generation. For a single isotropic plate, Bruno *et al.* demonstrated that, for a Gaussian beam, the optimum response is reached when the $1/e^2$ radius is $2\sqrt{2}/k$[4]. Extending this result for our bilayer system leads to an ideal pump radius of ∼4.6 µm for the first ZGV mode. In our set-up, detection sensitivity is inversely proportional to the probe beam radius. As both pump and probe beams are focused with the same objective lens (see Fig. 1(a) in the main text), it is difficult to achieve the ideal case. A good compromise is found with the pump and probe $1/e^2$ radii, measured by knife-edge technique, set to be 4.2 and 2.8 µm, respectively. This facilitates the generation of propagating modes with wavenumber $k = 0.67$ µm^{-1}, but modes in the range $0.3 \lesssim k \lesssim 1.4$ µm^{-1} should also be generated. In the case of the line pump spot with a $1/e^2$ intensity half-width of 1.5 µm and a length of 5 µm (used for the dispersion relation measurement), modes with wavenumbers in the range $0.8 \lesssim k \lesssim 3.9$ µm^{-1} are expected to be generated, as observed in experiment.

SUPPLEMENTARY NOTE 3: TEMPERATURE RISE EVOLUTION

The steady state temperature rise T of the sample at the centre of the optical pump spot is estimated by considering a finite-sized effectively 2D circular plate with its circumference held at constant temperature and approximating the laser intensity profile to a top hat distribution. Under such assumptions, the solution of the heat diffusion equation gives

$$T = \frac{P}{2\pi h\kappa} \times [\ln(a/w) + 1/2], \quad \text{(S7)}$$

where P is the power absorbed by the sample ($P = P_0 T_0 (1 - R_0)$ with $P_0 = 6$ mW the measured incident power before the objective lens, T_0=0.83 the optical transmittance of the objective lens at 415 nm—the pump wavelength—and R_0=0.444 the optical reflection coefficient of Ti at 415 nm), h the bilayer thickness (with $h_{\mathrm{Ti}} = 660$, $h_{\mathrm{Si_3N_4}} = 1830$ nm, see Supplementary Note 2), $\kappa = 27.8$ W.m^{-1}.K^{-1} the thermal conductivity estimated by weighting the values for each layer by their thickness ($\kappa_{\mathrm{Ti}} = 21.9$, $\kappa_{\mathrm{Si_3N_4}} = 30$ W.m^{-1}.K^{-1}), $a = 5.64$ mm the plate radius (the circular plate being chosen to have the same area as the square sample plate surface 10×10 mm^2) and $w = 4.2$ µm the $1/e^2$ intensity radius of the pump beam. Reflection coefficients and thermal conductivities are taken from Supplementary Ref. 5. It follows that T=49 K.

SUPPLEMENTARY REFERENCES

[1] J. D. Achenbach, *Wave propagation in elastic solids* (North-Holland, Amsterdam, 1984).

[2] A. Briggs, *Acoustic Microscopy*, pp: 102-103 (Clarendon, Oxford, 1992).

[3] R. D. Mindlin and J. Yang, *An introduction to the mathematical theory of vibrations of elastic plates* (World Scientific, 2006).

[4] F. Bruno, J. Laurent, P. Jehanno, D. Royer, and C. Prada, "Laser beam shaping for enhanced zero-group velocity Lamb modes generation", *J. Acoust. Soc. Am.*, **140**, 2829-2838 (2016).

[5] D. R. Lide, *CRC Handbook of Chemistry and Physics* (CRC-Press, 2010).
