# Supplementary material for: Imaging gigahertz zero-group-velocity Lamb waves
Source: Nat Commun. 2019 May 20;10:2228. doi: 10.1038/s41467-019-10085-4 (PMC6527571; doi:10.1038/s41467-019-10085-4)
Supplement: Supplementary file 2 — Description of Additional Supplementary Files [file 41467_2019_10085_MOESM2_ESM.pdf]

Description of Additional Supplementary Files

File Name: Supplementary Movie 1

Description: Imaging a ZGV Lamb mode. Normalized animations of the measured out-of-plane surface particle velocity at (a) 0.2391 and (b) 1.6900 GHz.
